# Draft genome sequence data of *Rhodosporidium toruloides* VN1, a strain capable of producing natural astaxanthin

**DOI:** 10.1016/j.dib.2019.104443

**Published:** 2019-08-28

**Authors:** Tuyet Nhung Tran, Dai-Hung Ngo, Ngoc Tuan Nguyen, Dai-Nghiep Ngo

**Affiliations:** a Department of Biochemistry, Faculty of Biology and Biotechnology, University of Science, Vietnam National University-HCM, Ho Chi Minh City, Viet Nam; b Faculty of Natural Sciences, Thu Dau Mot University, Binh Duong Province, Viet Nam; c Institute of Microbiology and Immunology, National Yang-Ming University, Taipei, Taiwan

**Keywords:** *Rhodosporidium*, Draft genome, Astaxanthin, Illumina

## Abstract

*Rhodosporidium toruloides* strain VN1 is of special interest because of its capability for high astaxanthin production. Here, we report the draft genome sequence of *R. toruloides* VN1, which comprises 20.01 Mb in 424 contigs with an overall G + C content of 61.8%. This whole-genome shotgun project has been deposited at DDBJ/EMBL/GenBank under the accession number SJTE00000000.

**Table:** Specifications Table

Subject	Biology
Specific subject area	Microbiology, Genomics
Type of data	Genomic sequence, gene prediction of *Rhodosporidium toruloides* VN1
How data were acquired	Whole genome was sequenced with an Illumina HiSeq. 2000 sequencing system
Data format	Raw sequencing reads, Draft genome assembly and gene prediction
Parameters for data collection	Genomic DNA from pure culture
Description of data collection	Whole genome shotgun sequencing followed by genome assembly and gene description
Data source location	*R. toruloides* VN1 was isolated from soil in Ho Chi Minh City, Vietnam
Data accessibility	This whole-genome shotgun project has been deposited at DDBJ/EMBL/GenBank under the accession number SJTE00000000 (https://www.ncbi.nlm.nih.gov/nuccore/SJTE00000000.1/). All raw sequence data have been deposited at NCBI Sequence Read Archive (SRA) under the accession number PRJNA525255 (https://www.ncbi.nlm.nih.gov/bioproject/PRJNA525255/).

**Table:** 

**Value of the data** • Draft genome data can provide a better understanding for astaxanthin production • Draft genome consist genes important for biotechnology • It will accelerate functional genomics research

## 1 Data

Astaxanthin (3,3′-dihydroxy-β-carotene-4,4′-dione) is mainly produced by chemical synthesis and has been widely used as a feed additive in the poultry and aquaculture industry [1], [2]. However, the chemical synthetic processes of astaxanthin negatively affect the environment and the use of synthetic astaxanthin raises the concern of food safety. To address these problems, production of natural astaxanthin from microorganisms has attracted considerable attention [1], [2]. *R. toruloides* VN1 that was isolated from soil in Vietnam was first used as a new microbial source for producing natural astaxanthin [2]. Here, we report the *R. toruloides* strain VN1 genome sequence, which can be used to explore the key genes in the astaxanthin production.

**Fig. 1:**
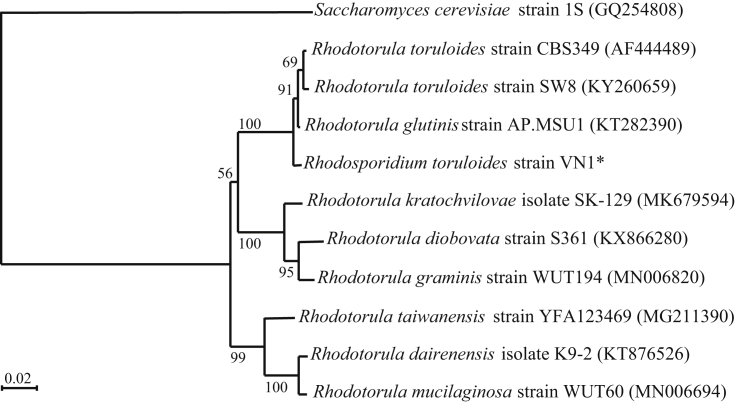
Phylogenetic neighbour-joining trees based on the nucleotide sequences of internal transcribed spacer (ITS)-region of isolated (indicated by star) and other species *Rhodosporidium* strains. The strains are indicated by their EMBL/GenBank/DDBJ accession numbers after species names. Bootstrap values, indicated at the nodes, are obtained from 1000 bootstrap replicates and are reported as percentages. Bar indicates 2% sequence divergence.

Illumina sequencing data generated 24.89 million paired-end reads with a total output of 2.51 Gb. The current draft comprises 424 contigs larger than 1000 bp in size, for a total size of 20,019,398 bp and a G + C content of 61.8% (Table 1). Overall, 8021 putative protein-coding genes and 126 tRNA have been identified. An internal transcribed spacer (ITS)-region phylogenetic tree based on Neighbour-Joining method places *R. toruloides* strain VN1 with other *R. toruloides* species (Fig. 1).

**Table 1:** Summary of the draft genome sequence of *Rhodosporidium toruloides* VN1.

No. of contigs	424
Total length	20,019,398 bp
Length of longest contig	455,949 bp
Mean length	47,215 bp
N50	108,558
GC content	61.8%
No. of predicted genes	8021
tRNA	126

## 2 Experimental design, materials, and methods

### 2.1 Genomic DNA preparation

*R. toruloides* strain VN1 was originally collected from soil samples in Ho Chi Minh City, Vietnam. Strain VN1 was inoculated in 50 ml of the basal medium (50 g l^−1^ sucrose, 10 g l^−1^ peptone, 3 g l^−1^ KH_2_PO_4_, and 3 g l^−1^ MgSO^4^, pH 6) and grown overnight at 30 °C with shaking at 200 rpm for 96 hours. The culture broth (50 mL) was centrifuged at 5000×*g* for 10 min at 4 °C. Total DNA was then extracted by using the I-genomic BYF DNA Extraction Mini Kit for fungi (iNtRON Inc., Seongnam, Korea). DNA concentration was measured by using a NanoDrop 2000с Spectrophotometer (Thermo Scientific) and then 500 ng/μL of genomic DNA was used for the sequencing.

### 2.2 Genome sequencing and assembly

Whole genome was sequenced by Theragen Etex Bio Institute (Republic of Korea) with the Illumina HiSeq 2000 platform using paired-end libraries with insert size of ∼100 bp. Approximately 2.51 Gb of raw data (101-bp reads with about 100 × sequencing depth) were generated. In order to perform quality trimming and adapter removal, pre-processing was carried out with the Trimmomatic tool using the following parameters: sliding window: 4:15; leading: 3; trailing: 3; minlength: 36 [3]. Quality assessment of the pre-processed data was performed using the FastQC tool version 0.11.8, which confirmed that poor quality bases were removed. *De novo* genome assembly was carried out with Velvet version 1.2.10 [4] and contigs with a length less than 200 bp were discarded to get reliable assembled results. The genes were predicted by GeneMark-ES [5], tRNAscan-SE [6] and BLAST.

### 2.3 Phylogeny analysis

The nucleotide sequences of internal transcribed spacer (ITS)-region from *R. toruloides* strain VN1 and the published strains were aligned using Clustal X (version 2.0.3). Using Bootstrap analysis with a default setting of 1000 trials and a seed value of 111, the phylogenetic tree was constructed.
